# An assessment of a pediatric early warning system score in severe hand-foot-and-mouth disease children: To detect clinical deterioration in hospitalized children

**DOI:** 10.1097/MD.0000000000011355

**Published:** 2018-06-29

**Authors:** Lu Mei, Xin Song, Yan Kong, Guiling Yu

**Affiliations:** a Qingdao Women and Children's Hospital; b Qingdao Municipal Center For Disease Control and Prevention; c Qingdao Institute of Preventive Medicine, Qingdao, P.R. China.

**Keywords:** early warning system, hand foot and mouth disease, pediatric

## Abstract

Identification of deteriorating severe hand, foot, and mouth disease (HFMD) children for referral to intensive care remains problematic.

The medical records of 2382 hospitalized children with severe HFMD from May 2013 to September 2015 were retrospectively reviewed. A Pediatric Early Warning System (PEWS) score was designed based on study parameters on admission, evaluated in a logistic regression model, and subsequently validated with different cut-off scores, to predict the risk for clinical deterioration.

After admission, 191 cases were transferred to the pediatric intensive care unit (PICU) and 2191 were admitted to the infectious disease department. Of which, 116 cases were subsequently transferred to PICU, with younger age, consciousness levels of sluggishness, lethargy or drowsiness, rashes with vesicles on the hands or feet, moderate or high fever, increased or disordered lung marking or pulmonary infiltration, abnormal heart rate, fasting plasma glucose, blood platelet, and C-reactive protein. A corresponding 10-component PEWS score >7 was significantly associated with subsequent transfer to PICU.

A 10-component PEWS score >7 has good specificity but poor sensitivity for identifying severe HFMD children vulnerable to clinical deterioration.

## Abstract 2

Supplemental Digital Content is available in the text

## 1 Introduction

Hand, foot, and mouth disease (HFMD) is a common acute infectious disease in children worldwide, featured by fever, painful sores in the mouth, and a rash with vesicles on the hands, feet, and buttocks,^[1]^ commonly caused by enteroviruses,^[2,3]^ and is mainly transmitted via the fecal-oral route, respiratory droplets, and contact with blister fluid with infected individuals. In China, since its first emergence in Shanghai in 1981,^[4]^ an unprecedented large-scale epidemic broke out in Anhui province in 2008.^[5]^ Severe cases, although rarely occurred among patients with HFMD, occasionally lead to encephalitis, aseptic meningitis, acute flaccid paralysis, pulmonary edema, pulmonary hemorrhage, myocarditis,^[6,7]^ or subsequent quick death.^[6,7]^ The dominant strain of HFMD is coxsackievirus A16 (CA16) and enterovirus 71 (EV71) worldwide^[7,8]^; however, severe cases with complications of central nervous system are always caused by EV71.^[6,7,9,10]^ Early recognition of deteriorating severe HFMD children with subsequent application of timely critical care services to match the severity of illness to an appropriate level of care, can significantly improve prognosis after those clinical deterioration.^[11–13]^

A Pediatric Early Warning System (PEWS) Score was developed, mainly for the pediatric emergency department with a need of admission to the pediatric intensive care unit (PICU), to rapidly assess the pediatric patient's status based on study parameters.^[12,14–17]^ Qingdao women and children's hospital is the designated hospital for treating severe HFMD in Qingdao municipality. Our study was aimed to design a score to identify deteriorating severe HFMD children through combining clinical and laboratory measures into a composite score and indicating by an increased likelihood of transfer to the PICU, and to permit the future development of score-matched care recommendations and aggressive therapy to minimize the impact exerted by severe HFMD.

## 2 Methods

### 2.1 Study population

The study was conducted at Qingdao women and children's hospital and in accordance with the principles of the Declaration of Helsinki, and approved by the institutional local committee on human research. The medical records of 2382 hospitalized children with severe HFMD from May 2013 to September 2015 in the infectious disease department at Qingdao women and children's hospital were retrospectively reviewed, including 1451 boys and 931 girls, aged 13 days to 16 years. Given the retrospective nature of this study and the use of anonymized patient data, requirements for informed consent were waived. Detailed patient information was abstracted from medical records including the clinical and laboratory measurements on admission and history of diseases.

### 2.2 Definition of severe HFMD case

According to diagnostic criteria defined by the National Health and Family Planning Commission of P.R. China, following the HFMD diagnosis and treatment guidelines (2008 Edition) (http://www.Nhfpc.gov.cn/zwgkzt/wsbysj/200812/38494.shtml), a case was defined as severe by the appearance of the symptoms/signs of HFMD in addition to more than one of the following complications: encephalitis, aseptic meningitis, acute flaccid paralysis, pulmonary edema, pulmonary hemorrhage, or myocarditis.

### 2.3 Clinical nursing classification

Patients were treated following the HFMD diagnosis and treatment guidelines (2008 edition) and cared according to clinical nursing classification criteria of “Principles of grading nursing in general hospital (Trial implementation)” (http://www.nhfpc.gov.cn/mohbgt/s9509/200905/40929.shtml), issued by Ministry of health of P.R. China in 2009, before May 1, 2014, and “WS/T 431-2013 Clinical nursing classification” (http://www.nhfpc.gov.cn/ewebeditor/uploadfile/2014/12/20141212142502408.PDF), issued by National Health and Family Planning Commission since then.

### 2.4 Measures of interest

PICU transfer was used as end point of clinically significant deterioration defined as involving failure in 1 or more systems including central nervous, circulatory, and respiratory system. During this study period, mortality was not observed in the patients.

### 2.5 Pediatric Early Warning System

In this study, a PEWS score was designed to assess a severe HFMD child's clinical status while hospitalized, based on study parameters, to predict the potential deterioration and the need for PICU transfer.

### 2.6 Statistical analysis

Twenty-seven candidate items available in our dataset were evaluated, including age; sex (male or female); temperature; level of consciousness (sober or conscious, sluggishness, lethargy, or drowsiness); heart rate (HR); fasting plasma glucose (FPG); white blood cell; blood platelet; hemoglobin; C-reactive protein (CRP); percentage of neutrophil granulocyte; blood pressure; capillary refill time (CRT); respiratory rate; respiratory effort; transcutaneous oxygen saturation; oxygen therapy; lung marking or pulmonary infiltration on chest radiograph; limbs tremor; rashes with vesicles on the hands, feet, mouth, or buttocks; papule; herpes; macula; and Babinski sign. Chi-square test was performed to compare distribution for categorical variables, between cases admitted to PICU and to the infectious disease department, and the Mann-Whitney *U* test was conducted to compare difference of means for continuous variables.

The logistic regression model was used for analysis of candidate variables in association with above binary outcomes. Akaike information criterion (AIC) or Bayesian Information Criterion (BIC) was used to judge the model fitness; the lower the AIC or BIC value the better the model fitness is. A Pediatric Early Warning System score was designed based on study parameters evaluated in a logistic regression model and subsequently validated with different cut-off scores, to predict the potential risk for subsequent clinical deterioration. The area under the receiver operating characteristics curve (AUCROC) was used to assess discrimination of different cut-off scores. Items with an AUCROC of ≤0.65 were excluded. A sensitivity analysis by including 191 admissions to PICU was performed for validation of the PEWS score. All analyses were performed with the software SPSS, version 17.0 and Stata, version 11.2.

## 3 Results

Among 2382 hospitalized patients, 191 cases were admitted to PICU and 2191 to the infectious disease department. Of which, 116 cases were transferred to PICU with subsequent clinical deterioration; with younger age; moderate or high fever; limbs tremor; rashes with vesicles on the hands or feet; positive Babinski sign; increased or disordered lung marking or pulmonary infiltration on chest radiograph; consciousness levels of sluggishness; lethargy or drowsiness; and abnormal HR, FPG, blood platelet, CRP, and CRT (*P* < .05, Table 1 and Supplemental Table S1). Enterovirus serotype was tested in 1215 (51%) individuals, 155 among those admitted to PICU, 1060 to the infectious disease department, with no significant difference (Pearson χ^2^ = 0.67, *P* = .31).

**Table 1:**
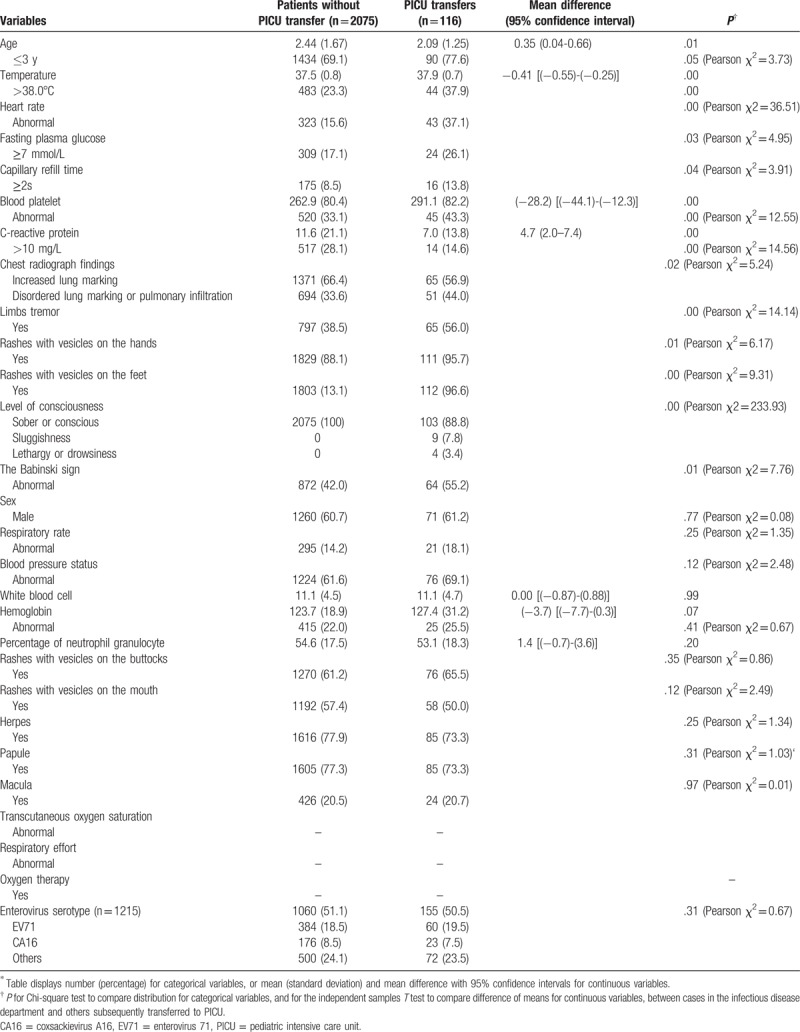
Sample characteristics of patients with or without the pediatric intensive care unit ^∗^transfer.

All of these significant elements were furthermore assessed in logistic regression model, as shown in Table 2, with characteristics of model fitness presented in Supplemental Table S2. Models with elements excluding the Babinski sign, CRT, or limbs tremor had smaller AIC or BIC than others, with no significant difference of model fitness to the complete model. In addition to consciousness levels, a “core” model with 9 dichotomous variables, including age, rashes with vesicles on the hands or feet, temperature, lung marking or pulmonary infiltration on chest radiograph, HR, FPG, blood platelet, and CRP had better model fitness.

**Table 2:**
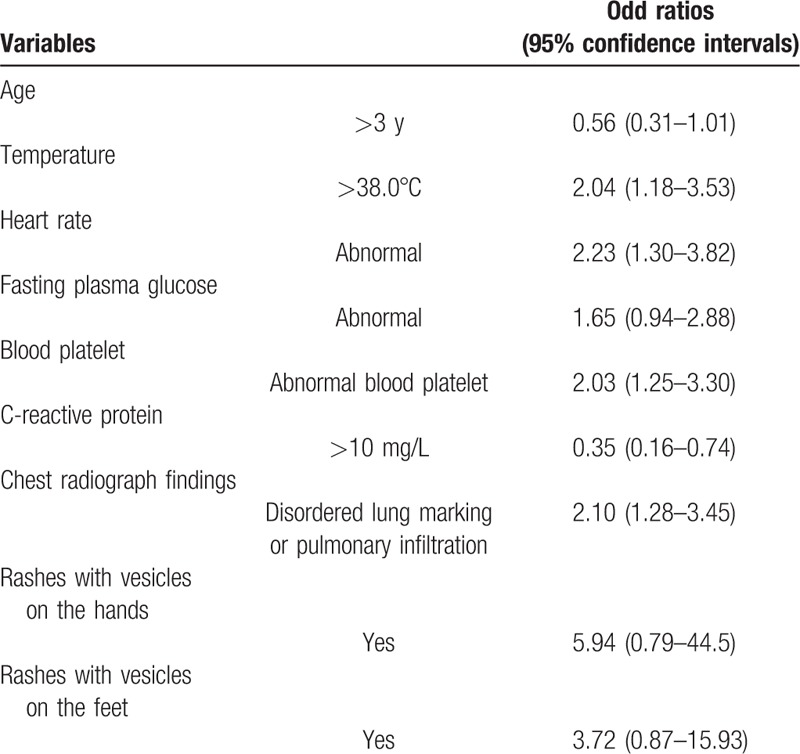
Odd ratios (95% confidence intervals) of deterioration of severe hand foot and mouth disease in association to baseline variables.

A PEWS score was designed accordingly based on these 10 study parameters, ranged 0 to 9 and subsequently validated with different cut-off scores, as shown in Table 3. AUROC for a cut-off PEWS score of 5, 6, and 7 was all higher than 0.65, with the lowest AIC or BIC in model with a cut-off PEWS score of 7. Patient with a PEWS score of 7 or higher was significantly associated with increased risk of PICU transfer (odd ratios [95% confidence intervals]: 9.10 [5.59–14.81] *P* = .00), compared with patient with a PEWS score of 0 to 6, and correctly identified 95.1% patients. In addition, model fitness of 13 items included with different cut-off PEWS scores was inferior to that in “core” model (Table 3). All PEWS scores were highly specific in this context (specificity 100%). A sensitivity analysis for validation of the Pews score further including 191 admissions to PICU was performed (Supplemental Table S3) and did not show any significant difference.

**Table 3:**
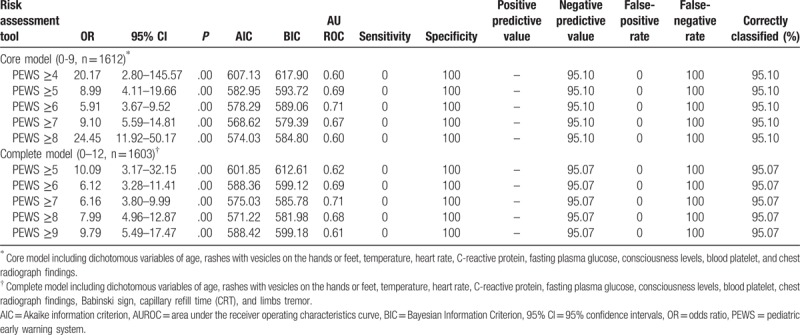
Performance characteristics of the Pediatric Early Warning System score among admissions to infectious diseases department.

## 4 Discussion

Severe HFMD patients with younger age; consciousness levels of sluggishness; lethargy or drowsiness; rashes with vesicles on the hands or feet; moderate or high fever; increased or disordered lung marking or pulmonary infiltration on chest radiograph; and abnormal HR, FPG, blood platelet, and CRP were associated with PICU transfer. Patients with a 10-component PEWS score of 7 or higher was significantly associated with increased risk of PICU transfer (odd ratio (95% confidence interval): 9.10 (5.59–14.81), *P* = .00) compared with patients with a PEWS score of 0 to 6, with 95.1% patients correctly identified.

Our study found that >84.3% of severe HFMD cases were in children younger than 4 years, in consistent with previous reports,^[18–20]^ which showed that young children had much higher probability of developing severe neurological complications.

Brainstem encephalitis was the most common neurologic complication by severe HFMD,^[21–29]^ characterized by a disturbance in the level of consciousness, such as lethargy, drowsiness, or coma,^[19,20]^ manifesting cytopathic damage to neuronal cells.^[23,24]^ Pulmonary edema or hemorrhage was considered to be an autonomic nervous system manifestation of brainstem encephalitis,^[30,31]^ seen as diffuse pulmonary infiltrates and congestion on a chest radiograph, which could be explained by immune enhancement resulting from superinfection by related enteroviruses infection by highly virulent strains.^[6,32]^ In addition, circulatory disturbance or even circulation failure is commonly observed in severe HFMD.^[29,33]^

Similarly, we found that severe cases were associated with appearance of oral lesions or ulcers,^[20,34]^ elevated white blood cell,^[19,34]^ or increased fasting blood glucose^[28]^ or CRP,^[35]^ which were suggested to be employed to diagnose severe HFMD at an earlier stage.

Severe HFMD inpatients were admitted to infectious diseases department with implementation of the first- or second-class nursing care, among whom, 116 cases were transferred to PICU with subsequent clinical deterioration, which suggests a number of children at significant risk of deterioration that are not being captured by physician assessment. Patients with elevated PEWS score are statistically more likely to be directly admitted or transferred to the PICU. Although PICU level of care is costly, earlier inclusion of borderline patients may extremely improve subsequent deterioration and optimize resource allocation in the hospital setting.^[11–13]^ Our results suggest that a PEWS score should be simultaneously tabulated by the nurse and integrated into physician communication to enhance the identification of deteriorating patients when admission, in particular, to assist with management of such severe patients, intensive or special clinical treatment, and care are recommended to be activated immediately if a PEWS score was elevated to be 7 or higher.

This PEWS score was designed based on study parameters to quantify severity of severe HFMD in hospitalized children. Patients were diagnosed and treated according to the HFMD diagnosis and treatment guidelines (2008 edition). So far, the causes of HFMD-related severe complications are still unclear. Recently, studies have found that EV 71 predominated among laboratory-confirmed severe and fatal cases,^[6,7,9,10]^ which were much more severe than CA16 infections. China has developed an inactivated EV71 vaccine, which is the first vaccine against EV71 in the world (National Health and Family Planning Commission of the People's Republic of China, http://www.nhfpc.gov.cn/qjjys/s3594r/201512/fa403581683d4b619bcee477aa15423e.shtml). In our study, a definitive diagnosis of severe HFMD was easily made for all admissions with typical complications observed and with type of virus causing severe HFMD not all available on admission, although of which would certainly to some extent add value to the model and should definitely look into it in further investigations.

Our study provides an objective methodology in exploration of optimum model, which is an algorithm-driven model selection in parameters and cut-off PEWS scores. Our study is, however, a retrospective cohort study, the PEWS score is designed based on data of medical records available on admission and lacks of type of virus causing HFMD, which might cause bias and limit the clinical practice. Only a few number of deteriorating severe HFMD cases was observed in each year, 41 in 2013, 58 in 2014, and 17 in 2015, respectively, validation of the score should be performed in our hospital or on an external cohort in future. The PEWS score was simplified using dichotomized parameters for ease of utility in clinical practice, prospective validation especially for the methodological rationality remains necessary to enhance the applicability. Moreover, further investigations on its medical care costs and the cost-effectiveness assessment are warranted. Numerous work still need to be done to further establish a comprehensive pediatric early warning system for clinical care.

Severe HFMD patients with a 10-component PEWS score of 7 or higher was significantly associated with increased risk of PICU transfer. A 10-component PEWS score higher than 7 has good specificity but poor sensitivity for identifying patients vulnerable to acute deterioration.

## Author contributions

**Conceptualization:** Lu Mei.

**Data curation:** Lu Mei, Guiling Yu.

**Formal analysis:** Xin Song.

**Investigation:** Lu Mei.

**Methodology:** Xin Song.

**Project administration:** Lu Mei, Guiling Yu.

**Resources:** Lu Mei.

**Software:** Xin Song.

**Supervision:** Guiling Yu.

**Validation:** Xin Song.

**Writing – original draft:** Lu Mei.

**Writing – review and editing:** Xin Song, Yan Kong.

## Supplementary Material

SUPPLEMENTARY MATERIAL
