# Supplementary material for: An assessment of a pediatric early warning system score in severe hand-foot-and-mouth disease children: To detect clinical deterioration in hospitalized children
Source: Medicine (Baltimore). 2018 Jun 29;97(26):e11355. doi: 10.1097/MD.0000000000011355 (PMC6039599; doi:10.1097/MD.0000000000011355)
Supplement: SUPPLEMENTARY MATERIAL [file medi-97-e11355-s001.docx]

Supplementary Table 1 Normal reference value of variables

| Item | Age group (year) | Normal Reference Value |
| --- | --- | --- |
| Respiratory rate | 0 to 1 | 90-160 |
|  | 1-5 | 90-140 |
|  | 5-12 | 70-120 |
|  | >12 | 60-100 |
| Blood pressure (mmHg) | 0 to 3 | SBP:60-100 / DBP:40-60 |
|  | 3-6 | SBP:70-110 /DBP:50-70 |
|  | 6-13 | SBP:80-120 /DBP:60-80 |
|  | >13 | SBP:100-140 /DBP:70-90 |
| Heart rate | 0 to 1 | 90-160 |
|  | 1-5 | 90-140 |
|  | 5-12 | 70-120 |
|  | >12 | 60-100 |
| C-reactive protein (mg/L) |  | ≤10 |
| Fasting plasma glucose (mmol/L) | | 3.9-7.0 |
| Capillary refill time (second) | | <2 |
| Transcutaneous oxygen saturation | | >94% |
| Blood platelet (×10^9^/L) |  | 100-400 |
| White blood cell (×10^9^/L) | | 4-10 |
| Neutrophilic granulocyte (%) | | 50-70 |
| Hemoglobin (g/L) | 0 to 1 | 170-200 |
|  | >1 | 110-160 |
| Level of consciousness |  | Sober or conscious |
| Babinski sign | 0 to 2 | Negative or bothside positive |
|  | >2 | Negative |

Abbreviations: SBP: systolic blood pressure; DBP: diastolic blood pressure.

Supplementary Table 2 Model fitness of logistic regression model

| Models | Number of observations | Degree of freedom | AIC | BIC | AUROC |
| --- | --- | --- | --- | --- | --- |
| Complete model (including 12 variables)* | 1599 | 13 | 555.47 | 625.37 | 0.78 |
| Complete model excluding age | 1599 | 12 | 557.82 | 622.35 | 0.77 |
| Complete model excluding rashes with vesicles on hands | 1599 | 12 | 557.90 | 622.43 | 0.77 |
| Complete model excluding rashes with vesicles on feet | 1599 | 12 | 557.67 | 622.19 | 0.78 |
| Complete model excluding temperature | 1599 | 12 | 559.75 | 624.28 | 0.77 |
| Complete model excluding heart rate | 1599 | 12 | 561.35 | 625.88 | 0.76 |
| Complete model excluding C-reactive protein | 1757 | 12 | 617.30 | 682.96 | 0.75 |
| Complete model excluding fasting plasma glucose | 1791 | 12 | 665.11 | 730.99 | 0.76 |
| Complete model excluding blood platelet | 1690 | 12 | 589.34 | 654.53 | 0.77 |
| Complete model excluding chest radiograph findings | 1603 | 12 | 561.86 | 626.42 | 0.76 |
| Complete model excluding Babinski sign | 1599 | 12 | 555.28 | 619.80 | 0.78 |
| Complete model excluding capillary refill time | 1604 | 12 | 554.02 | 618.58 | 0.78 |
| Complete model excluding limbs tremor | 1603 | 12 | 556.01 | 620.57 | 0.77 |
| Core model (including 9 variables)† | 1608 | 10 | 554.74 | 608.57 | 0.77 |

*Complete model with dichotomous variables of age, rashes with vesicles on the hands or feet, temperature, heart rate, C-reactive protein, fasting plasma glucose, blood platelet, chest radiograph findings, Babinski sign, capillary refill time (CRT) and limbs tremor.

†Core model with dichotomous variables of age, rashes with vesicles on the hands or feet, temperature, heart rate, C-reactive protein, fasting plasma glucose, blood platelet and chest radiograph findings.

AIC: Akaike’s information criterion; BIC: Bayesian Information Criterion; AUROC: area under the receiver operating characteristics curve.

Supplementary Table 3 Performance characteristics of the Pediatric Early Warning System (PEWS) score among 2382 admissions

| Risk assessment tool | OR | 95% CI | P | AIC | BIC | AU ROC | Sensitivity | Specificity | Positive predictive value | Negative predictive value | False-positive rate | False-negative  rate | Correctly classified (%) |
| --- | --- | --- | --- | --- | --- | --- | --- | --- | --- | --- | --- | --- | --- |
| Core model (0-9, n=1687)* | | | | | | | | |  |  |  |  |  |
| PEWS ≥ 4 | 4.72 | 2.29-9.72 | 0.00 | 1007.2 | 1018.1 | 0.58 | 0 | 100 | - | 90.87 | 0 | 100 | 90.87 |
| PEWS ≥ 5 | 3.77 | 2.48-5.71 | 0.00 | 986.9 | 997.7 | 0.64 | 0 | 100 | - | 90.87 | 0 | 100 | 90.87 |
| PEWS ≥ 6 | 3.80 | 2.71-5.32 | 0.00 | 976.2 | 987.0 | 0.65 | 0 | 100 | - | 90.87 | 0 | 100 | 90.87 |
| PEWS ≥ 7 | 6.28 | 4.27-9.25 | 0.00 | 960.5 | 971.3 | 0.63 | 0 | 100 | - | 90.87 | 0 | 100 | 90.87 |
| PEWS ≥ 8 | 17.28 | 9.10-32.84 | 0.00 | 965.1 | 975.9 | 0.58 | 16.23 | 98.89 | 59.52 | 92.16 | 1.11 | 83.77 | 91.35 |
| Complete model (0-12, n=1677)† | | | | | | | | | |  |  |  |  |
| PEWS ≥ 5 | 4.68 | 2.57-8.52 | 0.00 | 990.7 | 1001.5 | 0.60 | 0 | 100 | - | 90.88 | 0 | 100 | 90.88 |
| PEWS ≥ 6 | 5.12 | 3.34-7.85 | 0.00 | 956.0 | 966.9 | 0.67 | 0 | 100 | - | 90.88 | 0 | 100 | 90.88 |
| PEWS ≥ 7 | 7.64 | 3.79-9.99 | 0.00 | 935.0 | 945.9 | 0.71 | 0 | 100 | - | 90.88 | 0 | 100 | 90.88 |
| PEWS ≥ 8 | 6.66 | 4.61-9.62 | 0.00 | 938.3 | 949.2 | 0.65 | 0 | 100 | - | 90.88 | 0 | 100 | 90.88 |
| PEWS ≥ 9 | 7.64 | 4.73-12.3 | 0.00 | 971.2 | 982.0 | 0.59 | 0 | 100 | - | 90.88 | 0 | 100 | 90.88 |

*Core model including ten variables as described in Supplementary Table 2.

OR: odds ratio; 95%CI: 95% confidence intervals; AIC: Akaike’s information criterion; BIC: Bayesian Information Criterion; AUROC: area under the receiver operating characteristics curve.
